# The nucleic acid revolution continues – will forensic biology become forensic molecular biology?

**DOI:** 10.3389/fgene.2014.00044

**Published:** 2014-03-05

**Authors:** Peter Gunn, Simon Walsh, Claude Roux

**Affiliations:** ^1^Centre for Forensic Science, University of Technology SydneySydney, NSW, Australia; ^2^Forensics, Australian Federal PoliceCanberra, ACT, Australia

**Keywords:** forensic molecular biology, epigenetics, RNA, DNA, methylation

## Abstract

Molecular biology has evolved far beyond that which could have been predicted at the time DNA identity testing was established. Indeed we should now perhaps be referring to “forensic molecular biology.” Aside from DNA’s established role in identifying the “*who*” in crime investigations, other developments in medical and developmental molecular biology are now ripe for application to forensic challenges. The impact of DNA methylation and other post-fertilization DNA modifications, plus the emerging role of small RNAs in the control of gene expression, is re-writing our understanding of human biology. It is apparent that these emerging technologies will expand forensic molecular biology to allow for inferences about “*when*” a crime took place and “*what*” took place. However, just as the introduction of DNA identity testing engendered many challenges, so the expansion of molecular biology into these domains will raise again the issues of scientific validity, interpretation, probative value, and infringement of personal liberties. This Commentary ponders some of these emerging issues, and presents some ideas on how they will affect the conduct of forensic molecular biology in the foreseeable future.

## INTRODUCTION

The advent of forensic DNA testing not only revolutionized forensic science but also its contribution to investigations and court proceedings. It was also a key component in the elevation of the field from a niche science to a far reaching public-good science perceived very favorably by the general public. Notwithstanding this success, it is also necessary to highlight some challenges related to this development: exponential drain on resources, overemphasis on source attribution, related complex mathematical modeling and overemphasis on one single dimension of the informational content of a trace^1^. It is fair to say that, up to now, apart from its established role in identifying the “who” in criminal investigations and in Court, molecular biology played a relatively timid role in addressing other questions relevant to investigative and legal dimensions. A significant focus of development has been the sensitivity and discriminating power of core techniques, which, in the absence of concomitant research on the trace evidence properties themselves leads to the situation where DNA is often detected and profiled but no comment at all can be made about the nature of the biological material, or when or how it was deposited. However, recent developments in medical and developmental molecular biology are pushing the current frontiers of forensic applications and novel nucleic acid technologies are now ripe for application to expanded forensic challenges. This commentary presents some of these developments and illustrates how they may assist scientists to answer investigative, legal, or broader security questions in the future.

## CONTEXT AND PRESENT LIMITATIONS

The current applications of molecular biology to the investigation of crime have evolved over the past 25 years from the academic investigations of DNA structure and function, in particular that of non-coding DNA sequences (Jeffreys et al., 1985; Wolff et al., 1991). The predominant forensic application of molecular biology has been in human identity testing, using non-phenotypic markers such as restriction fragment length polymorphisms, mini- and micro- satellite sequences, and single nucleotide polymorphisms to identify the source of biological material. The choice of non-phenotypic markers for forensic analysis was driven primarily by their polymorphic diversity, coupled with the ethically acceptable lack of personal or medical information which they convey.

The analysis of these markers has become the cornerstone of forensic DNA testing, and will likely remain so for the foreseeable future, given their power of discrimination and the enormous financial and social investment that has been made worldwide in commissioning and supporting the technology and its analytical tools (databases, search engines, predictive software, etc.). There are many commercially produced DNA systems that are validated for forensic use; all are based on the analysis of panels of locus-specific microsatellite sequences known as short tandem repeats (STRs) by multiplex PCR, followed by capillary electrophoresis. See for example Hill et al. (2011) and the website of the National Institute of Standards and Technology (http://www.cstl.nist.gov/biotech/strbase/multiplx.htm) for an extensive (but by no means complete) catalog of available markers. Furthermore, these relatively stable DNA markers are transmitted by classical Mendelian genetics, which not only makes the analysis of parentage, kinship and population studies comparatively accessible to biologists, but is also in line with accepted biological models of inheritance.

When STR analysis is difficult or impossible due to the amount or quality of DNA that is recovered from an item, then examination of mitochondrial DNA (mtDNA) will sometimes be undertaken. Usually this is done by dideoxynucleotide (“Sanger”) sequencing of the hypervariable control region of the mitochondrial genome. While nowhere near as informative as STR analysis, mtDNA can provide useful investigative information or confirmation of identity in certain cases. The analysis of mtDNA in forensic biology has recently been reviewed by Holland et al. (2013).

STR markers are sufficiently polymorphic and amenable to multiplexing to allow for almost unambiguous identification of an individual, and so can place that person’s DNA at the scene of a crime with a high degree of certainty. However STRs do not convey to the investigator any information about:

• *when* or *how* that material was deposited,
• in the case of an unidentified source of the material, *what* cell/fluid/tissue source it came from, or
• any phenotypic descriptions of an otherwise anonymous person who left that material at the scene, other than their gender.

## RECENT DEVELOPMENTS IN MOLECULAR BIOLOGY

Advances in other areas of molecular biology over the past decade have revealed new levels of information contained within the nucleic acids, at both the organism and cellular level, far beyond that of simple DNA structure and sequence. There are several variants of such expression, which are often grouped together as “epigenetics”:

They can be *RNA-mediated*, in which for example, tissue-specific micro-RNA (miRNA) sequences, usually about 20–25 bases long, influence the expression of genes, via their interaction with messenger RNA (mRNA). See for example Lagos-Quintana et al. (2002), wherein it is postulated that the population of expressed miRNAs plays a role in tissue specification or cell lineage decisions.

Specific sequences of *DNA* (predominantly the cytosine bases in runs of CpG sequences) become methylated during the course of an organism’s development. The sites of methylation are specific to chromosomal location, and to a subset of cells or tissues, and assert extensive control of the expression of genes in those cells. Methylation of specific sites can be governed by behavioral or other environmental influences (Feinberg, 2007; Goldberg et al., 2007). Intriguingly, methylated patterns of DNA can be passed on to offspring (Zhao et al., 2005), which has profound implications for such established paradigms as Mendelian and Darwinian inheritance.

Specific methylation patterns are associated with particular disease states and other phenotypic traits, and can be detected in the laboratory by variants of current classical and next-generation DNA sequencing technologies (Madi et al., 2012).

Thus these emerging technologies have implications not only for the medical sciences, (see for example Bell and Spector, 2011), but of particular significance to the authors of this Commentary is the application of these technologies to forensic biology. Methylation epigenetics is well suited to the detection and identification of body fluids, exploiting the differential methylation of specific chromosomal sites between tissues (Madi et al., 2012) and between individuals, even including identical twins (Li et al., 2011). Similarly, the tissue-specific expression of miRNAs can be used for the identification of body fluids in a forensic setting (Zubakov et al., 2010a; Courts and Madea, 2011).

In a forensic context, these advances in molecular biology have already shown potential in the identification of tissue types, and a demonstrated role in various behavioral traits (although *cause and effect* need to be demonstrated). For relevant examples, see references Petronis et al. (2000) and Boulle et al. (2012).

## THE SUITABILITY OF METHYLATED DNA AND miRNAs IN FORENSIC APPLICATIONS

As Zubakov et al. (2010a) state: “MicroRNAs (miRNAs) are non-protein coding molecules with important regulatory functions; many have tissue-specific expression patterns. Their very small size in principle makes them less prone to degradation processes, unlike messenger RNAs (mRNAs), which were previously proposed as molecular tools for forensic body fluid identification.”

Furthermore, the use of either miRNAs or methylated DNA to identify body fluids has the advantage that extraction and purification methods are compatible with existing DNA purification methods; thus one extraction and purification process can provide templates both for body fluid identification and classical DNA identification (Madi et al., 2012). In contrast, traditional biochemical and serological body fluid identification techniques are often destructive, and not always compatible with downstream DNA processing (Raymond et al., 2011).

## DETERMINING THE ORIGIN OF BIOLOGICAL SPECIMENS

Considerable research has been undertaken into trying to establish the source of cells, fluid or tissue by its specific messenger RNA profile. When DNA has been isolated and characterized from unidentified body material, such as “touch” specimens, it can become a matter of courtroom contention as to the nature of that deposit – for example was it from sweat, or saliva or some other fluid? While gross deposits can be identified, there is a need for the forensic scientist to be able to characterize specimens that are increasingly smaller in size, and are from other than the “traditional” sources of DNA, so that the origin of this material can be contextualized. Many practitioners have explored the use of fluid-specific messenger RNA profiles to perform this characterization (Juusola and Ballantyne, 2005; Hanson et al., 2009, 2012; ). However there is an overarching concern about using mRNAs, due to the inherent instability of messenger RNA (Haas et al., 2011).

In contrast miRNAs are more stable than mRNAs, in particular more resistant to degradation, and hence are better able to survive a range of crime scene conditions (Hanson et al., 2009; Madi et al., 2012). Investigations of panels of miRNAs to date have concentrated on the identification of the most forensically common tissues/fluid such as blood, semen, and saliva but references to other tissue-specific miRNAs are available in the literature (Pai et al., 2011).

Likewise the origin of tissues and biofluids can be determined with a high degree of certainty by DNA of specific methylated DNA sequences (Madi et al., 2012).

By applying these emerging technologies in conjunction with “classical” DNA identification techniques (STRs, mtDNA), the forensic scientist may exploit the informational content of a biological trace beyond that of simple *identification* of a donor, to a more holistic exploitation that that may also support inferences of *activity*.

## ESTIMATING THE AGE OF SPECIMEN DONOR

Individual age is one of the major factors determining human appearance. Establishing the age of an unknown person may provide important leads in police investigations, disaster victim identification, identity fraud cases, or in determining whether to try defendants as adults or juveniles. Currently used methods of age determination rely mostly on odontological or anthropological analysis. These techniques require the availability of human remains such as teeth, bones, or even the whole body. They are also subject to wide tolerances in terms of the conclusions and variation across different population groups. The development of molecular methods for age estimation using specimens that possess no phenotypic information, e.g., bloodstains, is of practical value, as these types of traces commonly occur at the crime scene.

Predicting age by molecular means has been achieved at the research level by several techniques, however none of these precursor methods has the resolution of accuracy to stand alone as a reliable predictive tool.

These techniques include the analysis of T-cell receptor re-arrangements (“signal joint TCP excision circles” – sjTRECs) which has shown that the decline in the number of these in body fluids (primarily but not exclusively blood) is a reasonably good predictor of age (Zubakov et al., 2010b; Xue-ling et al., 2012). Standard real-time PCR techniques are readily adaptable to quantify this predictor.

Likewise the telomeres in peripheral leucocytes have been shown to shorten in an age-dependent manner (Ren et al., 2009), again as measured by standard molecular biological techniques such as Southern blotting.

Similarly, the analysis of the methylation patterns in the promoters of the Edar-Associated Death Domain (EDARADD), TOM1L1 (a gene coding for a protein of unknown function), and Neuronal Pentraxin II (NPTX2) genes is linear with age over a range of five decades (Bocklandt et al., 2011).

## PREDICTING PHENOTYPIC CHARACTERISTICS OF SPECIMEN DONOR

Considerable progress has been made in predicting phenotypic characteristics of the specimen donor (other than their gender). Single nucleotide polymorphisms (SNPs) are being exploited to predict phenotypic characteristics (also referred to as externally visible characteristics – EVCs) such as skin pigmentation, eye color, and biogeographic origin. These have been extensively reviewed elsewhere (Kayser, 2011). There have recently been introduced into the forensic community SNP tests that claim to predict with considerable certainty eye color (Irisplex®), (Walsh et al., 2011), and both eye and hair color (Hirisplex® Walsh et al., 2013). Implementation of these into casework is still problematic, and there continues to be extensive disquiet in the wider judicial community about the ethical and legal implications of these applications (Koops and Maurice, 2008), in particular the potential for misapplication as a “racial profiling” tool. (M’charek et al., 2012) contend that “*…questions about defining populations … and the application of EVCs in criminal investigation – lie at the core of most social, ethical, and legal issues raised by the translation of EVCs into forensic and police practices*.” There are also more practical challenges that limit the adoption of these genetic technologies such as the availability of a stable technological product (akin to the commercially produced equivalents in routine use), the level of understanding within mainstream forensic institutions of deeper scientific and bio-ethical issues, an operational framework where such applications can be effective in delivering technical intelligence to investigators, and the capacity of forensic experts to articulate and advocate issues that impact the effectiveness of the applications.

## CONCLUDING COMMENTS

We have presented here just a few examples of the potential for new molecular biology technologies to assist in the investigation of crime. It has been our casework experience that there is frequent need for supporting evidence, beyond the identification of source, particularly where trace DNA deposits are pivotal components of a circumstantial case. Just as the introduction of DNA identity testing prompted a number of scientific, moral and legal challenges, so the expansion of molecular biology into these domains will raise again the issues of scientific validity, interpretation, probative value, and infringement of personal liberties. It is, however, hoped that the forensic science, law enforcement and legal communities have now more experience on how to deal with such issues than when forensic DNA profiling originally came to the fore. The development and fostering of forensic science as a distinctive holistic discipline (Margot, 2011; Roux et al., 2012) and the establishment of a stronger research culture in forensic science (Mnookin et al., 2011) will also assist to achieve a relatively smooth transition.

Will these or other technologies make their way into the crime lab? Possibly not; they are specialized, and are not likely to be called upon often enough to warrant the financial and logistical commitment that would be required of an operational forensic lab. But where the expertise to undertake these tests exists in other research settings such as universities, then we foresee the day when these academies will be called upon to lend their expertise to forensic investigations.

As forensic DNA profiling technologies such as rapid DNA increase the potential of DNA 1 day being applied as another modality of biometrics, the role of forensic institutions will focus more extensively on the full exploitation of crime scene material. In this sense, modern forensic molecular biology will only be as good as it allows scientists to answer investigative, legal or broader security questions. Doing this requires new science and new knowledge and this review provides an insight into opportunities to deliver both.

## Conflict of Interest Statement

The authors declare that the research was conducted in the absence of any commercial or financial relationships that could be construed as a potential conflict of interest.

## Footnotes

1 The term “trace” is applied here to any mark, material or remnant of an activity or presence. It is used independently from the actual size of this remnant (Margot, 2011).
